# Interaction of Polymerase Subunit PB2 and NP with Importin α1 Is a Determinant of Host Range of Influenza A Virus

**DOI:** 10.1371/journal.ppat.0040011

**Published:** 2008-02-01

**Authors:** Gülsah Gabriel, Astrid Herwig, Hans-Dieter Klenk

**Affiliations:** Institute of Virology, Philipps University Marburg, Germany; University of Wisconsin-Madison, United States of America

## Abstract

We have previously reported that mutations in the polymerase proteins PB1, PB2, PA, and the nucleocapsid protein NP resulting in enhanced transcription and replication activities in mammalian cells are responsible for the conversion of the avian influenza virus SC35 (H7N7) into the mouse-adapted variant SC35M. We show now that adaptive mutations D701N in PB2 and N319K in NP enhance binding of these proteins to importin α1 in mammalian cells. Enhanced binding was paralleled by transient nuclear accumulation and cytoplasmic depletion of importin α1 as well as increased transport of PB2 and NP into the nucleus of mammalian cells. In avian cells, enhancement of importin α1 binding and increased nuclear transport were not observed. These findings demonstrate that adaptation of the viral polymerase to the nuclear import machinery plays an important role in interspecies transmission of influenza virus.

## Author Summary

The natural hosts of influenza A viruses are aquatic birds. On rare occasions these viruses may be transmitted to humans and then give rise to an influenza pandemic. Human influenza is therefore a typical re-emerging infection. Evidence is increasing that the viral polymerase, an enzyme that has to enter into the nucleus of the infected cell in order to promote replication and transcription of the viral genome, is a major determinant of host range. Thus, in a comparative study of an avian influenza strain and its mouse adapted variant we have previously shown that adaptation to mice depended exclusively on mutations in the polymerase proteins. These findings supported the concept that adaptation of the polymerase to host factors is an important mechanism underlying interspecies transmission. In the present study, we have identified importin α1, a component of the nuclear pore complex, as such a host factor. We show that adaptive mutations in polymerase subunits improve binding to importin α1 in mammalian, but not in avian cells. As a result, nuclear transport of these proteins and efficiency of replication are enhanced in mammalian cells. These observations demonstrate that the interaction of the viral polymerase with the nuclear import machinery is an important determinant of host range.

## Introduction

The natural host of influenza A viruses is waterfowl where these agents occur in large variety defined by 16 hemagglutinin and 9 neuraminidase subtypes. Avian influenza viruses are the source of devastating outbreaks in poultry. Moreover, because of their potential to cross species barriers, to adapt to new hosts, and to cause on rare occasions pandemics, they are also a constant threat to human health [1]. As members of the *Orthomyxoviridae* family influenza A viruses have a segmented RNA genome of negative polarity. The eight segments are present in enveloped virus particles as ribonucleoprotein (RNP) complexes with the nucleocapsid protein (NP) and the three subunits of the RNA-dependent RNA-polymerase (PB1, PB2, PA). In the infected cell, the polymerase is responsible for transcription and replication of the viral genome [2,3].

Evidence is increasing that the viral polymerase plays a major role in host adaptation. Thus, in a comparative study of the avian strain SC35 (H7N7) and its mouse-adapted variant SC35M we showed that adaptation to mice was the result of seven mutations in the polymerase proteins, six of which (L13P and S678N in PB1, D701N and S714R in PB2, K615N in PA, and N319K in NP) were responsible for enhanced polymerase activity in mammalian cells. In avian cells, replication and transcription of SC35M were reduced, whereas both activities were increased with SC35. Furthermore, the differences in polymerase activity were paralleled by differences in pathogenicity: SC35 was highly pathogenic for chickens and had reduced pathogenicity in mice, whereas the opposite was the case with SC35M. Thus, the efficiency of the viral polymerase is a determinant of both host specificity and pathogenicity [4,5]. PB2 mutation D701N has also been implicated in the adaptation of H5N1 viruses to mammalian hosts [6,7] but other mutations may be involved, too, notably PB2 mutation E627K [8–13]. All of these findings support the concept that adaptation of the polymerase to host factors is an important mechanism underlying interspecies transmission [5].

The influenza virus polymerase is active in the nucleus of the infected cell where cytoplasmicly expressed PB1 and PA appear to be imported as a subcomplex which then assembles with separately imported PB2 [14]. There is evidence that the different polymerase subunits use the classical import pathway of the host cell depending on the recognition of a nuclear localization signal (NLS) of the cargo protein by an importin α/β dimer as well as non-classical pathways that rely on direct interaction of the cargo with an importin β homologue receptor [15]. Thus, PB1-PA dimers enter the nucleus via a non-classical transport pathway by binding to RanBP5 [16]. NP, in contrast, binds to importin α1/α2 [17] indicating that it uses the classical pathway. The same route is used for the nuclear transport of PB2 as demonstated by a recent crystallographic study showing that the C-terminus of this protein forms a complex with importin α5 [18]. Interestingly, the authors of this study hypothesized that PB2 mutation D701N which is also a host range determinant as pointed out above, may affect the binding of importin α5 to PB2. These observations suggest that importins belong to the host factors to which the polymerase adapts during interspecies transmission.

To test this hypothesis we have compared now in avian and mammalian cells the interaction of the polymerase complex of SC35 and SC35M with importin α1, which not only binds to NP, but is also the most abundant importin in a variety of human cells [19] and may function as an interchangeable housekeeping transport factor [20]. We show that adaptative mutations D701N and N319K improve binding of PB2 and NP, respectively, to importin α1 in mammalian, but not in avian cells. As a result, the efficiency of the transport of these proteins into the nucleus of mammalian cells is enhanced. These data support the notion that the interaction of PB2 and NP with importin α1 plays an important role in determining host range and pathogenicity of influenza A viruses.

## Results

### Binding of PB2 and NP to Importin α1 in Mammalian and Avian Cells

**Figure 1:**
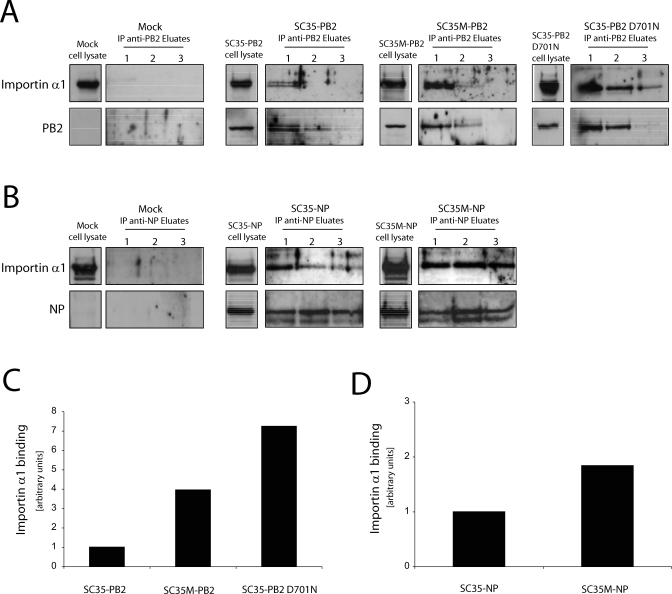
Interaction of PB2 and NP with Importin α1 in Mammalian Cells 293T cells were transfected with plasmids encoding SC35-PB2 (30 μg), SC35M-PB2 (30 μg), and SC35-PB2 D701N (30 μg) (A) and SC35-NP (10 μg) and SC35M-NP (10 μg) (B) proteins. As a control, non-transfected cells were used (Mock). 48 h after transfection cells were harvested and immunoprecipitated using the respective PB2 or NP antibody. Precipitated eluates (1–3) and non-precipitated whole cell lysates of transfected cells were subjected to SDS gel electrophoresis, and PB2, NP, and importin α1 were detected by Western blot analysis. In (B) two NP bands can be seen of which the minor one most likely represents a cleavage product [39]. The total amount of importin α1 bound in all three eluates to PB2 (C) and NP (D) was quantitated using Image J software (http://rsb.info.nih.gov/ij/). An arbitrary unit is the amount of importin α1 bound to SC35 PB2 and SC35 NP, respectively.

**Figure 2:**
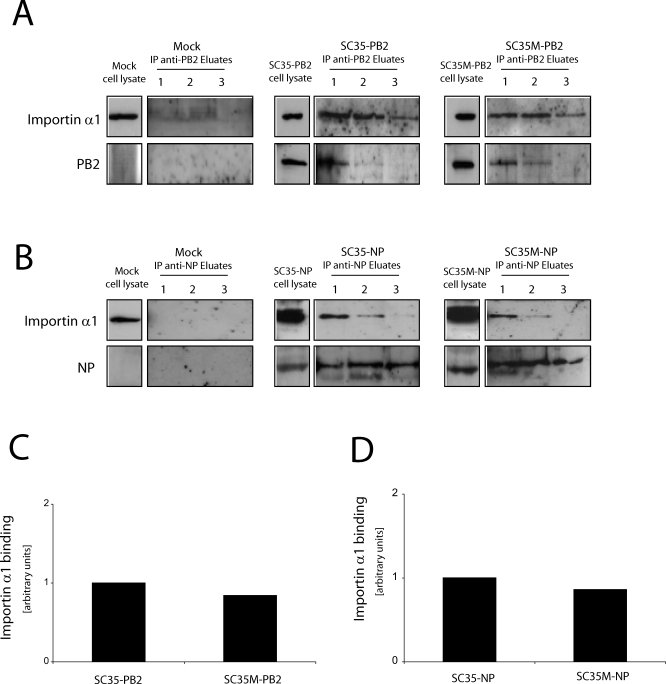
Interaction of PB2 and NP with Importin α1 in Avian Cells CEC-32 cells were transfected with plasmids encoding SC35-PB2 (30 μg), SC35M-PB2 (30 μg), and SC35-PB2 D701N (30 μg) (A) and SC35-NP (10 μg) and SC35M-NP (10 μg) (B) proteins. As a control, non-transfected cells were used (Mock). 48 h after transfection cells were harvested and immunoprecipitated using the respective PB2 or NP antibody. Precipitated eluates (1–3) and non-precipitated whole cell lysates of transfected cells were subjected to SDS gel electrophoresis, and PB2, NP, and importin α1 were detected by Western blot analysis. Importin α1 bound to PB2 (C) and NP (D) was quantitated as described in the legend of Figure 1.

To test the hypothesis that the polymerase proteins of SC35 and SC35M differ in their interaction with the nuclear import machinery of mammalian and avian cells we have analyzed the binding of PB2 and NP to importin α1 in co-immunoprecipitation experiments. Cultures of human 293T cells and quail embryo cells (CEC-32) were transfected with plasmids encoding PB2 or NP. Importin α1 complexed with PB2 or NP was precipitated from lysates of transfected and mock transfected cells using specific antibodies against PB2 or NP, which were covalently coupled to an amine-reactive gel. Bound proteins were eluted three times from the columns using acidic elution buffer to allow quantitative estimation of interaction partners. All three eluates were used to detect importin α1, PB2 and NP, respectively, by Western blot analysis. In 293T cells transfected with PB2 of SC35M or PB2 D701N, 4 to 7 times more importin α1 was bound than in cells transfected with PB2 of SC35 (Figure 1A and 1C). Likewise, NP derived from SC35M precipitated 2 times more importin α1 from 293T cells than SC35 NP (Figure 1B and 1D). In contrast, SC35 PB2 and SC35M PB2 bound similar amounts of importin α1 when CEC-32 cells were transfected (Figure 2A and 2C). There was also no difference in importin binding efficiency when SC35 NP and SC35M NP were expressed in avian cells (Figure 2B and 2D). These observations indicate that the adaptive mutations PB2 D701N and NP N319K specifically improve the binding of PB2 and NP to mammalian importin α1 adaptor protein.

### Subcellular Localization of Importin α1 in Mammalian and Avian Cells

**Figure 3:**
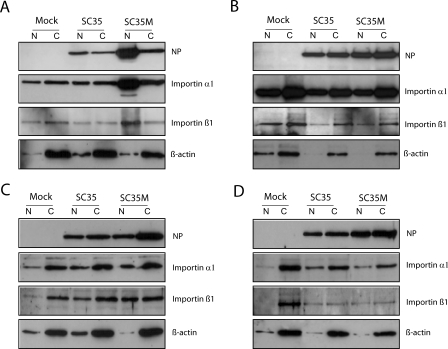
Importin α1 Distribution in Mammalian and Avian Cells 293T or CEC-32 cells were infected at MOI 2 with SC35 or SC35M. 293T cells were harvested 6 h p.i. (A) and 12 h p.i. (B), and CEC-32 cells were harvested 9 h p.i. (C) and 18 h p.i. (D) and separated into nuclear and cytoplasmic fractions. NP, importin α1, importin ß1, and ß-actin were determined by Western blot analysis.

**Figure 4:**
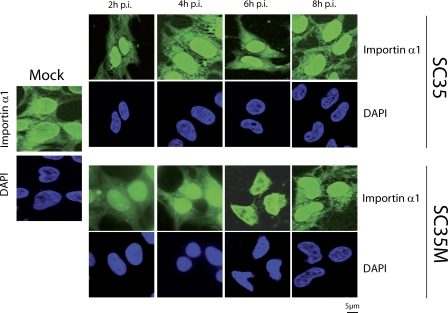
Kinetics of Nuclear Transport of Importin α1 293T cells were infected at MOI 2 with SC35 and SC35M, and the localization of human importin α1 was determined by immunofluorescence assays at different time points after infection (2 h p.i., 4 h p.i., 6 h p.i., and 8 h p.i.). For detection of importin α1, FITC-coupled secondary antibody was used. Cell nuclei were stained with DAPI.

It was now of interest to find out if increased binding of NP and PB2 to importin α1 affected nuclear transport in mammalian cells. First, we have carried out Western blot experiments to determine importin α1 in nuclear and cytoplasmic fractions prepared from infected cells early and late in the replication cycle. Since replication kinetics were different in avian and mammalian cells, time points with comparable virus titres were selected (6 h and 12 h p.i. in 293 T cells, 9 h and 18 h p.i. in CEC32 cells) (Figure S1). Early after infection of 293T cells with SC35M, we found a distinct accumulation of importin α1 as well as importin ß1 and NP in the nucleus. This phenomenon was not observed with SC35 (Figure 3A). Late in infection nuclear accumulation of these proteins was seen neither with SC35 nor with SC35M (Figure 3B). Nuclear accumulation of importin α1 was also not detected in avian cells early or late after infection with either virus. Furthermore, the expression levels of SC35 and SC35M NP were similar in the nuclear and cytoplasmic fractions of CEC-32 cells at both time points (Figure 3C and 3D). When unfractionated cell lysates were analyzed, no differences in the amounts of importin α1 were detected, irrespective of cell type, virus strain, and time after infection (Figure S2). This observation indicates that the increased amount of importin α1 seen in the nuclei of 293T cells early after infection with SC35M was the result of increased transport into the nucleus and did not reflect up-regulated importin α1 synthesis. We have then determined the kinetics of nuclear accumulation of importin α1. 293T cells were infected with SC35M or SC35, and the intracellular localization of importin α1 was determined at different intervals by immuno-fluorescence analysis (Figure 4). In cells infected with SC35M, importin α1 was gradually shifted from the cytoplasm to the nucleus where it was almost exclusively present 6 h p.i. Later in infection it was detected again in the cytoplasm. In cells infected with SC35, importin α1 was equally distributed between cytoplasm and nucleus throughout the replication cycle. Taken together, these results demonstrate that SC35M induces specifically in mammalian cells a transient accumulation of importin α1 in the nucleus with a peak at 6 h p.i. which presumably coincides with the maximal replication rate of the virus.

### Effect of Adaptive PB2 and NP Mutations on the Localization of Importin α1 in Mammalian Cells

**Figure 5:**
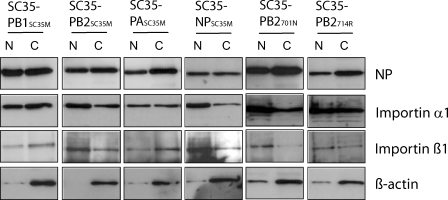
Distribution of Importin α1 in Mammalian Cells 293T cells were infected at MOI 2 with SC35, SC35M, SC35-PB1_SC35M_, SC35-PB2_SC35M_, SC35-PA_SC35M_, SC35-NP_SC35M_, SC35-PB2_701N_, and SC35-PB2_714R_. Cells were harvested 6 h p.i. and separated into nuclear and cytoplasmic fractions. NP, importin α1, importin ß1, and ß-actin were determined by Western blot analysis.

To identify the mutations in the polymerase proteins of SC35M responsible for the nuclear accumulation of importin α1, we have first analyzed single gene reassortant (SGR) viruses containing one of the polymerase genes of SC35M in a SC35 background. Cells infected with SGR viruses were separated into nuclear and cytoplasmic fractions, and importin α1 and importin ß1 localization was determined (Figure 5). Only SGR viruses SC35-PB2_SC35M_ and SC35-NP_SC35M_ containing the PB2 or the NP gene of SC35M showed enhanced importin α1 and importin ß1 levels in the nuclear fractions, although the increase was not as distinct as with the parental SC35M virus (Figure 3). With the SGR viruses containing PB1 or PA of SC35M, there was no importin accumulation in the nucleus (Figure 5). Since SC35M NP differs from SC35 NP only by one amino acid substitution (N319K), it can be concluded that this mutation contributes to nuclear accumulation of importin. SC35 PB2 displays two amino acid substitutions contributing to increased mouse virulence (D701N and S714R). To determine which of these mutations controls nuclear transport of importin, we infected cells with single point mutant (SPM) viruses SC35-PB2_701N_ and SC35-PB2_714R_ containing only one of these mutations. Nuclear accumulation of importin α1 was observed in SC35-PB2_701N_ infected, but not in SC35-PB2_714R_ infected cells (Figure 5). These findings indicate that mouse adaptation mutations PB2 D701N and NP N319K promote nuclear accumulation of importin α1 in mammalian cells.

### Transport of PB2 and NP Subunits in Mammalian Cells

**Figure 6:**
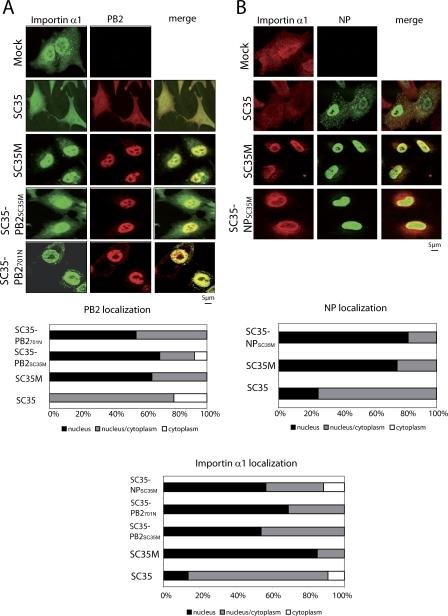
Localization of PB2, NP, and Importin α1 in Mammalian Cells by Immunofluorescence Analysis A549 cells were infected with SC35, SC35M, SC35-PB2_SC35M_, and SC35-PB2_701N_ (MOI 2). 6 h p.i. cells were stained with antisera specific for importin α1 (secondary antibody FITC-coupled) and PB2 (secondary antibody Rhodamine-coupled) (A) or importin α1 (secondary antibody Rhodamine-coupled) and NP (secondary antibody FITC-coupled) (B). The amounts of PB2, NP, and importin α1 in the nucleus and the cytoplasm are indicated by bars. Quantification was done as described in Materials and Methods.

Some of the data described so far suggested already that nuclear accumulation of importins in SC35M infected mammalian cells is accompanied by increased NP transport into the nucleus. To further analyze the effect of the adaptive mutations in NP and PB2 on the intracellular localization of these proteins, we have performed immunofluorescence assays in mammalian cells. While PB2 of SC35M was predominantly located in the nucleus of human lung cells (A549), most of the cells infected with SC35 showed a nuclear and cytoplasmic distribution of PB2 (Figure 6A). This observation indicates that SC35M PB2 is transported into the nucleus more efficiently than SC35 PB2. Increased nuclear transport of SC35M PB2 correlated with the nuclear accumulation of importin α1 as indicated by a distinct co-localization of PB2 and importin α1 in the nucleus. On the other hand, in cells infected with SC35, importin α1 was present throughout cytoplasm and nucleus as was the case with PB2 (Figure 6A). When A549 cells were infected with SGR virus SC35-PB2_SC35M_ and SPM virus SC35-PB2_701N_, PB2 was also concentrated in the nucleus where it co-localized with importin α1 (Figure 6A). Nuclear accumulation of PB2 was not observed in cells infected with SC35-PB2_714R_ (data not shown). We have then analyzed the nuclear import of NP by immunofluorescence (Figure 6B). NP of SC35M and SC35-NP_SC35M_ was located predominantly in the nucleus, whereas a large part of SC35 NP was found in the cytoplasm. In cells infected with SC35M and SC35-NP_SC35M_, importin α1 was concentrated in the perinuclear region where it partly co-localized with NP. In SC35 infected cells importin α1 prevailed in the cytoplasm. Similar results have been obtained when the intracellular localization of these proteins was analyzed by immunofluorescence in 293T human kidney cells (Figure S3). Taken together, these observations indicate that, in mammalian cells, mutations PB2 D701N and NP N319K enhance the efficiency of the nuclear import of PB2 and NP as well as importin α1.

### Nuclear Transport of PB2 and NP in Avian Cells

**Figure 7:**
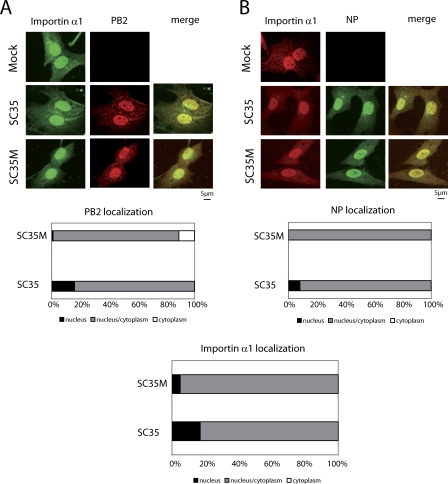
Localization of PB2, NP, and Importin α1 in Avian Cells CEC-32 cells were infected with SC35 and SC35M at MOI 2. 12 h p.i. cells were stained with antisera specific for importin α1 (secondary antibody FITC-coupled) and viral PB2 (secondary antibody Rhodamine-coupled) (A) or importin α1 (secondary antibody Rhodamine-coupled) and NP (secondary antibody FITC-coupled) (B). The amounts of PB2, NP, and importin α1 in the nucleus and the cytoplasm are indicated by bars. Quantification was done as described in Materials and Methods.

Intracellular localization of NP, PB2 and importin α1 has also been analyzed in CEC-32 cells. With both viruses, PB2 was clearly present in the nucleus, but it was also detected in the cytoplasm. Importin α1 co-localized with PB2 in the cytoplasm as well as the nucleus (Figure 7A). NP displayed a similar intracellular distribution pattern. Again, there was co-localization with importin α1 in the nucleus and in the cytoplasm, and there were no differences between SC35 and SC35M (Figure 7B). Thus, it appears that PB2 and NP of the avian and the mouse-adapted virus are transported with the same efficiency into the nucleus of avian cells.

## Discussion

We show here that mutations D701N in PB2 and N319K in NP responsible for mouse adaptation of the avian influenza virus SC35 enhance binding of these proteins to importin α1 of mammalian origin and, thus, improve the efficiency of their transport into the nucleus of mammalian cells. Increased transcription and replication activities in mammalian cells [4,5] are therefore, at least in part, the result of facilitated recruitment of polymerase subunits into the nucleus. In avian cells, neither enhancement of importin binding and nuclear transport nor increased polymerase activity [5] were observed. The differences in the nuclear transport of PB2 and NP were reflected by the efficiency of virus replication. In human lung cells SC35M grew to virus titres 2 logs higher than SC35. The SGR virus SC35-NP_SC35M_ grew as well as SC35M, whereas titres of SGR virus SC35-PB2_ SC35M_ were slightly reduced. In avian cells, parental and SGR viruses grew to equally high titres (Figure S5). These findings demonstrate that adaptation of PB2 and NP to importin α1 plays an important role in interspecies transmission. It has to be pointed out, however, that adaptive mutations different from PB2 D701N and NP N319K have been described in SC35M as well as numerous other viruses [4,6,7,21,22]. Thus, host adaptation is clearly a multifactoral process.

Importin α is the receptor for NLS-bearing cargo proteins. PB2 was first shown to contain NLSs at residues 449–495 and 736–739 [23]. The recent crystallographic analysis by Tarendeau and coworkers (2007) revealed that the latter sequence was part of a classical bipartite NLS comprising residues 738–755 which has to be unfolded by release of a salt bridge between aspartate 701 and arginine 753 to allow binding to importin α5. These authors also suggested that the adaptive mutation D701N observed in PB2 of SC35M might affect binding to importin α5 in different species. By showing that this mutation, indeed, improves importin α binding and nuclear transport of PB2 in mammalian cells we now confirm this concept. It has to be pointed out, however, that importin α5 used for the crystallographic analysis and importin α1 identified as a binding partner of PB2 in the present study differ significantly from each other in tissue specificity and cargo selectivity [24,25]. There are also large variations in amino acid sequence, but tryptophan residues 149, 191, 234 and 360 in importin α5, shown to be directly involved in the binding of the bipartite NLS of PB2 [18], are highly conserved and present in importins α5 as well as α1 (Figure S4) indicating that the crystallographic data obtained with importin α5 are also relevant for importin α1. However, additional structural and functional studies are clearly needed to assess the role of individual members of the importin α group in host adaptation. More detailed structural analysis of PB2 binding to importin α1 may also help to explain why adaptive PB2 mutation S714R did not affect in our study importin α1 binding and nuclear transport, even though it has also been implicated in PB2 binding to importin α5 [18].

Considerably less is known about the mechanism by which mutation N319K improves importin α1 binding and nuclear transport of NP in mammalian cells. Several NLSs have been identified on NP. An unconventional NLS located between amino acid residues 1–13 was shown to be involved in NP binding to importin α1 and importin α2 [17]. The second NLS is a bipartite signal located in the middle of NP between residues 198–216 which contributes less to the nuclear localization of NP than the unconventional NLS at the amino-terminus [26]. Furthermore, a nuclear accumulation site has been attributed to residues 336–345 in microinjection studies employing Xenopus oocytes and cells of rodent and primate origin [27,28]. None of these sites includes amino acid 319. A mutation in this position may therefore modulate one of the NLSs identified on NP by an allosteric mechanism.

Interestingly, we did not see differences in importin α1 binding and nuclear localization of PB2 and NP when we compared SC35 and SC35M in avian cells. Thus, while up-regulating nuclear transport in mammalian cells, the adaptive mutations did not interfere with it in avian cells albeit 82% homology between the avian and the human importin α1 (data not shown). This observation supports the concept that the virus when crossing the species barrier, goes through a phase that allows gradual acquisition of adaptive mutations without loosing fitness for the old host [5].

NLSs have also been identified in PB1 [29] and PA [30], but there is no apparent link between these signals and adaptation mutations L13P and S678N in PB1 and K615N in PA. Furthermore, neither importin binding nor nuclear localization of PB1 and PA have been analyzed in the present study, but the observation that PB1 and PA of SC35M did not induce nuclear accumulation of importin α1 (Figure 5) is compatible with the view that these proteins enter the nucleus via a non-classical pathway [16]. Altogether, however, it remains to be seen whether the adaptive mutations observed in PB1 and PA up-regulate polymerase activity in mammalian cells also by increased nuclear transport or by another mechanism. All cellular proteins using the classical nuclear import pathway have to compete with each other for importin α, and there is evidence that the equilibrium of these transport events which is necessary for the functional integrity of the cell is unbalanced when nuclear accumulation of importin α is excessive. This has been observed under various stress conditions, such as heat shock, UV irradiation and oxidative stress [31,32]. The cytoplasmic depletion of importin α1 as occurring in SC35M infected mammalian cells may therefore interfere with the nuclear transport of cellular proteins needed for the initiation of an antiviral status or for other vital functions and, thus, represent a new pathogenetic mechanism.

## Materials and Methods

### Cells and viruses.

293T (human embryonic kidney cells) and A549 (human lung carcinoma) cells were grown in DMEM (Dulbecco's minimal essential medium) supplemented with 10% FCS (fetal calf serum; Gibco). CEC-32 quail embryo fibroblasts [33] were grown in RPMI-1640 (Gibco) supplemented with 5% FCS and 2% chicken serum (Sigma). Influenza A viruses were propagated in 11 day old embryonated chicken eggs. Growth curves were determined in three independent experiments by plaque titration [34]. The recombinant viruses SC35 and SC35M and their mutants have been described before. Briefly, the chicken adapted virus SC35 was originally derived from the seal isolate A/Seal/Mass/1/80 (H7N7) by 35 passages in chicken embryo cells. SC35M was obtained from SC35 by sequential passages in mouse lung [35,36].

### Virus infection and transfection.

Monolayers of 293T, A549 and CEC-32 cells were infected with recombinant virus and incubated for 30 min for absorption at 37°C. Cells were washed twice with phosphate-buffered saline (PBS) and incubated for 6 h, 12 h and 18 h at 37°C in appropriate medium containing 0.2% bovine serum albumin (MP Biomedicals). For transfection, we seeded the cells in 100-mm-dishes and used 10μg pHW2000-SC35-NP, 10 μg pHW2000-SC35M-NP, 30 μg pHW2000-SC35-PB2, or 30 μg pHW2000-SC35M-PB2 plasmid [4] using Lipofectamine^TM^ 2000 (Invitrogen) according to the manufacturers protocols. The medium was replaced with fresh growth medium at 6 h posttransfection and incubated for further 48 h for immunoprecipitation assays.

### Growth curves.

293T, A549 and CEC-32 cells were inoculated with virus at a multiplicity of infection (MOI) of 2 for single cycle replication and at an MOI of 10^−4^ for multicycle replication. Virus inoculum was removed after 30 min of incubation at 37°C, and cells were washed two times with PBS pH5. Cells were then incubated in the appropriate medium containing 0.2% bovine serum albumin at 37°C. At time points 0, 3, 6, 9, 12, 15, 18, and 24 h for single cycle replication and at time points 0, 24, 48, 72, and 96 h for multicycle replication, we collected supernatants and determined plaque titers on MDCK cells. The growth curves shown are the average result of two independent experiments.

### Cell fractionation and western blotting.

60-mm-dishes of 293T and CEC-32 cells were infected with a MOI of 2. At the indicated time points, cells were washed twice with PBS and fractionated in 1ml PBS by using a Mixermill MM301 homogenizer (Retsch) at 20 Hz for 20 min. Lysates were mixed with 1% NP40 and separated into a nuclear (bottom) and a cytoplasmic (upper) fraction by centrifugation on a 20% sucrose cushion at 3000 rpm for 20 min. Nuclear fractions were sonicated for 15 min. Whole lysates and cell fractions were subjected to SDS-polyacrylamide gel electrophoresis followed by Western blot analysis as described [37]. Monoclonal antibodies specific for importin α1 (BD Biosciences) were used to detect the importin α1 distribution. As an internal standard, β-actin was determined with specific antibodies (Abcam). The results shown are representatives of three independent experiments.

### Immunofluorescence assay.

293T, A549 or CEC-32 cells were grown on glass cover slips and infected at a MOI of 2 with recombinant virus. At the indicated time points after infection, cells were fixed with PBS containing 2% paraformaldehyde and permeabilized with PBS containing 0.1% Triton-X-100. After blocking fixation with 3% BSA in PBS, we incubated the cells for 1h with monoclonal antibodies specific for importin α1 (BD Biosciences), PB2 (Santa Cruz) or NP (kindly provided by M. Schwemmle, Freiburg). After incubation with primary antibodies we washed the cells three times with PBS and incubated for further 30 min with either FITC-coupled goat anti-rabbit (1/200; Jackson ImmunoResearch Laboratories), FITC-coupled goat anti-mouse (1/200; Jackson ImmunoResearch Laboratories), Rhodamine-coupled donkey anti-goat (1/200; Jackson ImmunoResearch Laboratories) or Rhodamine-coupled goat anti-mouse (1/200; Jackson ImmunoResearch Laboratories) secondary antibodies. Cells were washed three times with PBS and coverslips were mounted on glass plates. Cells were observed in an Axiovert 200M microscope equipped with an ApoTome device (Zeiss). Localization of importin α1, PB2, and NP in nucleus, nucleus/cytoplasm and cytoplasm was determined using the microscope by counting cells (*n* = 100) infected with recombinant viruses. The results shown represent three independent experiments.

### Co-immunoprecipitation assay.

Transfected cells were washed twice with PBS and collected by centrifugation. Cell pellets were resuspended in 1 ml PBS and sonicated for 15 min. Complexes of importin α1 and viral proteins were determined by co-immunoprecipitation using the ProFound^TM^ Co-Immunoprecipitation Kit (PIERCE) according to the protocols provided by the manufacturer. Briefly, the antibodies specific for PB2 (Santa Cruz) or NP (polyclonal serum raised against A/FPV/Rostock/34 (H7N7) [37]) were coupled covalently to an amine-reactive gel (PIERCE) using the provided coupling buffer (0.14M sodium chloride, 0.008M sodium phosphate, 0.002 potassium phosphate, 0.01M KCl; pH7.4). Cell lysates were added to the antibody-coupled columns and incubated with gentle end-over-end mixing for 2 h at room temperature. Columns were then washed several times with coupling buffer to remove non-specifically bound material and finally eluted using the provided elution buffer (pH2.8). Protein complexes generally eluted in the first three fractions. Eluted immunoprecipitation complexes were then separated by SDS-polyacrylamide gel electrophoresis followed by Western blot analysis. For detection of the PB2 protein in Western blot we used the monoclonal anti-PB2 antibody which was kindly provided by Juan Ortin, Madrid [38]. For the detection of the NP protein we used polyclonal serum raised against A/FPV/Rostock/34 (H7N7). The results shown represent two independent experiments.

## Supporting Information

Figure S1 Growth Curves of SC35 and SC35M in 293T and CEC-32 Cells 293T (A) and CEC-32 (B) cells were infected at MOI 2. Virus titers were determined in cell culture media by plaque assay at different time points.
(383 KB EPS)

Figure S2 Expression Levels of Importin α1, Importin β1, and Viral Proteins in Mammalian and Avian Cells 293T cells were infected with SC35 and SC35M (MOI 2) and harvested 6 h p.i. (A) and 12 h p.i. (B). CEC-32 cells were infected with SC35 and SC35M (MOI 2) and harvested 12 h p.i. (C) and 18 h p.i. (D). NP, M1, importin α1, and importin β1 were detected in whole cell lysates by Western blotting.
(1.4 MB EPS)

Figure S3 Localization of PB2, NP, and Importin α1 in Mammalian Cells by Immunofluorescence Analysis 293T cells were infected with SC35, SC35M, SC35-PB2_SC35M_, and SC35-PB2_701N_ (MOI 2) and stained 6 h p.i. with antisera specific for PB2 (secondary antibody Rhodamine-coupled) and importin α1 (secondary antibody FITC-coupled) (A) or NP (secondary antibody FITC-coupled) and importin α1 (secondary antibody Rhodamine-coupled) (B).
(2.1 MB EPS)

Figure S4 Sequence Alignment of Human Importin α1, Importin α3, Importin α4, Importin α5, Importin α6, and Importin α7 by ClustalW (2.8 MB EPS)

Figure S5 Multicycle Replication in A549 and CEC-32 Cells A549 (A) and CEC-32 (B) cells were infected with SC35, SC35M, SC35-PB1_SC35M_, SC35-PB2_SC35M_, SC35-PA_SC35M_, and SC35-NP_SC35M_ at an MOI 0.0001, and virus titers in the supernatant were determined by plaque assay at different time points.
(424 KB EPS)

## References

[ppat-0040011-b001] Klenk HD, Matrosovich M, Stech J, Mettenleiter TC, Sobrino F (2007). Avian influenza: molecular mechanisms of pathogenesis and host range. Animal viruses: molecular biology.

[ppat-0040011-b002] Elton D, Digard P, Tiley L, Ortin J, Kawaoka Y (2006). Structure and function of influenza virus RNP. Influenza virology: current topics.

[ppat-0040011-b003] Neumann G, Brownlee GG, Fodor E, Kawaoka Y (2004). Orthomyxovirus replication, transcription, and polyadenylation. Curr Top Microbiol Immunol.

[ppat-0040011-b004] Gabriel G, Dauber B, Wolff T, Planz O, Klenk HD (2005). The viral polymerase mediates adaptation of an avian influenza virus to a mammalian host. Proc Natl Acad Sci U S A.

[ppat-0040011-b005] Gabriel G, Abram M, Keiner B, Wagner R, Klenk HD (2007). Differential polymerase activity in avian and mammalian cells determines host range of influenza virus. J Virol.

[ppat-0040011-b006] Li Z, Chen H, Jiao P, Deng G, Tian G (2005). Molecular basis of replication of duck H5N1 influenza viruses in a mammalian mouse model. J Virol.

[ppat-0040011-b007] Salomon R, Franks J, Govorkova EA, Ilyushina NA, Yen HL (2006). The polymerase complex genes contribute to the high virulence of the human H5N1 influenza virus isolate A/Vietnam/1203/04. J Exp Med.

[ppat-0040011-b008] Fouchier RA, Schneeberger PM, Rozendaal FW, Broekman JM, Kemink SA (2004). Avian influenza A virus (H7N7) associated with human conjunctivitis and a fatal case of acute respiratory distress syndrome. Proc Natl Acad Sci U S A.

[ppat-0040011-b009] Hatta M, Gao P, Halfmann P, Kawaoka Y (2001). Molecular basis for high virulence of Hong Kong H5N1 influenza A viruses. Science.

[ppat-0040011-b010] Labadie K, Dos Santos Afonso E, Rameix-Welti MA, van der Werf S, Naffakh N (2007). Host-range determinants on the PB2 protein of influenza A viruses control the interaction between the viral polymerase and nucleoprotein in human cells. Virology.

[ppat-0040011-b011] Naffakh N, Massin P, Escriou N, Crescenzo-Chaigne B, van der Werf S (2000). Genetic analysis of the compatibility between polymerase proteins from human and avian strains of influenza A viruses. J Gen Virol.

[ppat-0040011-b012] Puthavathana P, Auewarakul P, Charoenying PC, Sangsiriwut K, Pooruk P (2005). Molecular characterization of the complete genome of human influenza H5N1 virus isolates from Thailand. J Gen Virol.

[ppat-0040011-b013] Subbarao EK, London W, Murphy BR (1993). A single amino acid in the PB2 gene of influenza A virus is a determinant of host range. J Virol.

[ppat-0040011-b014] Fodor E, Smith M (2004). The PA subunit is required for efficient nuclear accumulation of the PB1 subunit of the influenza A virus RNA polymerase complex. J Virol.

[ppat-0040011-b015] Mattaj IW, Englmeier L (1998). Nucleocytoplasmic transport: the soluble phase. Annu Rev Biochem.

[ppat-0040011-b016] Deng T, Engelhardt OG, Thomas B, Akoulitchev AV, Brownlee GG (2006). Role of ran binding protein 5 in nuclear import and assembly of the influenza virus RNA polymerase complex. J Virol.

[ppat-0040011-b017] Wang P, Palese P, O'Neill RE (1997). The NPI-1/NPI-3 (karyopherin alpha) binding site on the influenza a virus nucleoprotein NP is a nonconventional nuclear localization signal. J Virol.

[ppat-0040011-b018] Tarendeau F, Boudet J, Guilligay D, Mas PJ, Bougault CM (2007). Structure and nuclear import function of the C-terminal domain of influenza virus polymerase PB2 subunit. Nat Struct Mol Biol.

[ppat-0040011-b019] Kohler M, Fiebeler A, Hartwig M, Thiel S, Prehn S (2002). Differential expression of classical nuclear transport factors during cellular proliferation and differentiation. Cell Physiol Biochem.

[ppat-0040011-b020] Goldfarb DS, Corbett AH, Mason DA, Harreman MT, Adam SA (2004). Importin alpha: a multipurpose nuclear-transport receptor. Trends Cell Biol.

[ppat-0040011-b021] de Jong MD, Simmons CP, Thanh TT, Hien VM, Smith GJ (2006). Fatal outcome of human influenza A (H5N1) is associated with high viral load and hypercytokinemia. Nat Med.

[ppat-0040011-b022] Finkelstein DB, Mukatira S, Mehta PK, Obenauer JC, Su X (2007). Persistent host markers in pandemic and H5N1 influenza viruses. J Virol.

[ppat-0040011-b023] Mukaigawa J, Nayak DP (1991). Two signals mediate nuclear localization of influenza virus (A/WSN/33) polymerase basic protein 2. J Virol.

[ppat-0040011-b024] Quensel C, Friedrich B, Sommer T, Hartmann E, Kohler M (2004). In vivo analysis of importin alpha proteins reveals cellular proliferation inhibition and substrate specificity. Mol Cell Biol.

[ppat-0040011-b025] Friedrich B, Quensel C, Sommer T, Hartmann E, Kohler M (2006). Nuclear localization signal and protein context both mediate importin alpha specificity of nuclear import substrates. Mol Cell Biol.

[ppat-0040011-b026] Weber F, Kochs G, Gruber S, Haller O (1998). A classical bipartite nuclear localization signal on Thogoto and influenza A virus nucleoproteins. Virology.

[ppat-0040011-b027] Davey J, Colman A, Dimmock NJ (1985). Location of influenza virus M, NP and NS1 proteins in microinjected cells. J Gen Virol.

[ppat-0040011-b028] Davey J, Dimmock NJ, Colman A (1985). Identification of the sequence responsible for the nuclear accumulation of the influenza virus nucleoprotein in Xenopus oocytes. Cell.

[ppat-0040011-b029] Nath ST, Nayak DP (1990). Function of two discrete regions is required for nuclear localization of polymerase basic protein 1 of A/WSN/33 influenza virus (H1 N1). Mol Cell Biol.

[ppat-0040011-b030] Nieto A, de la Luna S, Barcena J, Portela A, Valcarcel J (1992). Nuclear transport of influenza virus polymerase PA protein. Virus Res.

[ppat-0040011-b031] Furuta M, Kose S, Koike M, Shimi T, Hiraoka Y (2004). Heat-shock induced nuclear retention and recycling inhibition of importin alpha. Genes Cells.

[ppat-0040011-b032] Miyamoto Y, Saiwaki T, Yamashita J, Yasuda Y, Kotera I (2004). Cellular stresses induce the nuclear accumulation of importin alpha and cause a conventional nuclear import block. J Cell Biol.

[ppat-0040011-b033] Zoller B, Redman-Muller I, Nanda I, Guttenbach M, Dosch E (2000). Sequence comparison of avian interferon regulatory factors and identification of the avian CEC-32 cell as a quail cell line. J Interferon Cytokine Res.

[ppat-0040011-b034] Stech J, Xiong X, Scholtissek C, Webster RG (1999). Independence of evolutionary and mutational rates after transmission of avian influenza viruses to swine. J Virol.

[ppat-0040011-b035] Li SQ, Orlich M, Rott R (1990). Generation of seal influenza virus variants pathogenic for chickens, because of hemagglutinin cleavage site changes. J Virol.

[ppat-0040011-b036] Scheiblauer H, Kendal AP, Rott R (1995). Pathogenicity of influenza A/Seal/Mass/1/80 virus mutants for mammalian species. Arch Virol.

[ppat-0040011-b037] Wagner R, Herwig A, Azzouz N, Klenk HD (2005). Acylation-mediated membrane anchoring of avian influenza virus hemagglutinin is essential for fusion pore formation and virus infectivity. J Virol.

[ppat-0040011-b038] Barcena J, Ochoa M, de la Luna S, Melero JA, Nieto A (1994). Monoclonal antibodies against influenza virus PB2 and NP polypeptides interfere with the initiation step of viral mRNA synthesis in vitro. J Virol.

[ppat-0040011-b039] Zhirnov OP, Konakova TE, Garten W, Klenk H (1999). Caspase-dependent N-terminal cleavage of influenza virus nucleocapsid protein in infected cells. J Virol.

